# Hospital Saturday Fund

**Published:** 1892-08-20

**Authors:** Reginald B. D. Acland

**Affiliations:** Chairman Hospital Saturday Fund. 8, King's Bench Walk, Temple, E.C.


### HOSPITAL SATURDAY FUND.

Sir,—My attention has only this evening been directed to your "Passing Topics" of July 30th. I have hitherto found it impossible to correct all "Agricola's" misrepresentations with regard to the Hospital Saturday Fund, but pray allow me, with regard to the article in question, sufficient space to say that, so far from "twenty-four hundred ladies" having "between them collected £1,902," the street collection this year amounted in round figures to £5,500. The sum mentioned by "Agricola" was collected in a few districts only; the number of ladies mentioned approximately represents those collecting in all the districts. Though we should be happy to give "Agricola" any information he may require if he will ask for it, I am almost afraid it is too much to ask him to be more careful in future to ascertain what has been done before he "flatters himself that if he had 2,400 zealous ladies at command" he would do so very much better.—Yours faithfully,

Reginald B. D. Acland,
Chairman Hospital Saturday Fund.

8, King's Bench Walk, Temple, E.C.,

August 10th, 1892.

[The observations offered upon the Saturday Fund Street Collection were not conceived in an unkindly spirit. The sum of £1,902 was given in the daily papers, morning and evening, as the total collection, and although it may be urged that a correction of the figures at the time they appeared would have been more to the purpose, it is nothing but gratifying to learn, even thus late, that the 2,400 ladies achieved greater things than were chronicled.—Agricola.]

